# Veno-venous extracorporeal membrane oxygenation-assisted treatment of severe airway stenosis due to goiter: Two case reports

**DOI:** 10.1097/MD.0000000000039506

**Published:** 2024-09-06

**Authors:** Shuang-Long Zhang, Wang Zheng, Qi-Feng Zhang, Shi-Lei Zhao, Gang Li, Wei Sun, Li-Na Meng, Wuyuntana Han, Hong-Xun Yuan

**Affiliations:** a Department of Critical Care Medicine, Peking University International Hospital, Beijing, China; b Department of Neurosurgery, Tianjin Medical University General Hospital, Tianjin, China; c Department of Radiology, Peking University International Hospital, Beijing, China; d Horqin Zuoyi Zhongqi Mongolian Medicine Hospital, Inner Mongolia, China.

**Keywords:** airway stenosis, goiter, VV-ECMO

## Abstract

**Rationale:**

Extracorporeal membrane oxygenation (ECMO) is a critical care intervention that acts as a temporary substitute for the heart and lungs, facilitating adequate tissue perfusion and gas exchange. The 2 primary configurations, veno-arterial and veno-venous ECMO, are tailored to support either the heart and lungs or the lungs alone, respectively.

**Patient concerns:**

The case report details patients with tumor-induced airway stenosis who encountered limitations with standard treatments, which were either insufficient or carried the risk of severe complications such as hypoxia and asphyxia.

**Diagnoses:**

Patients were diagnosed with severe airway stenosis caused by goiter, a condition that required innovative treatment approaches to prevent complications during the management process.

**Interventions:**

Veno-venous ECMO was implemented as a bridging therapy to provide vital respiratory support during the tumor resection procedure. This intervention was crucial in reducing the risks associated with airway edema or tumor rupture.

**Outcomes:**

With the use of veno-venous ECMO, the patients successfully underwent tumor resection. They were subsequently weaned off the ECMO support, and after a course of treatment, they were discharged in good condition.

**Lessons:**

The case demonstrates the efficacy of veno-venous ECMO as a bridging therapy for managing severe airway stenosis caused by goiter. Its use facilitated the successful resection of tumors and led to positive patient outcomes, highlighting its potential as a valuable treatment option in similar scenarios.

## 1. Introduction

Goiter commonly invades the larynx, trachea, and esophagus, and surgical resection is the main treatment in such cases. Due to the goiter compressing the airway, airway stenosis and breathing difficulty are already present preoperatively, and there is a high risk of life-threatening situations such as airway edema leading to suffocation and asphyxia.^[1,2]^ For such patients, it is impossible to establish an effective airway through the supraglottic method, as endotracheal intubation can easily cause airway edema, tumor bleeding, or even the tumor falling off and blocking the airway, leading to suffocation. In this report, we describe the treatment of 2 such cases. Following the preoperative discussion among the multidisciplinary ECMO team consisting of members from the general surgery, otorhinolaryngology, anesthesiology, and Intensive Care Unit departments, the patients were diagnosed with severe airway stenosis. This was an indication for the use of veno-venous extracorporeal membrane oxygenation (VV-ECMO) treatment, and the patients underwent tumor resection and airway reconstruction under VV-ECMO assistance. Both patients were successfully weaned from the machine after surgery and showed a good short- and long-term prognosis.

## 2. Medical details

### 2.1. Case 1

The patient was a 70-year-old woman weighing 78 kg who presented to the hospital with “a 10-year-old thyroid nodule, hoarseness, and dyspnea for one month.” The patient was diagnosed with hypothyroidism and found to have thyroid nodules 10 years ago but did not undergo regular medical treatment. She had hoarseness with dyspnea in the past month and then was admitted to the hospital.

**Figure 1.**
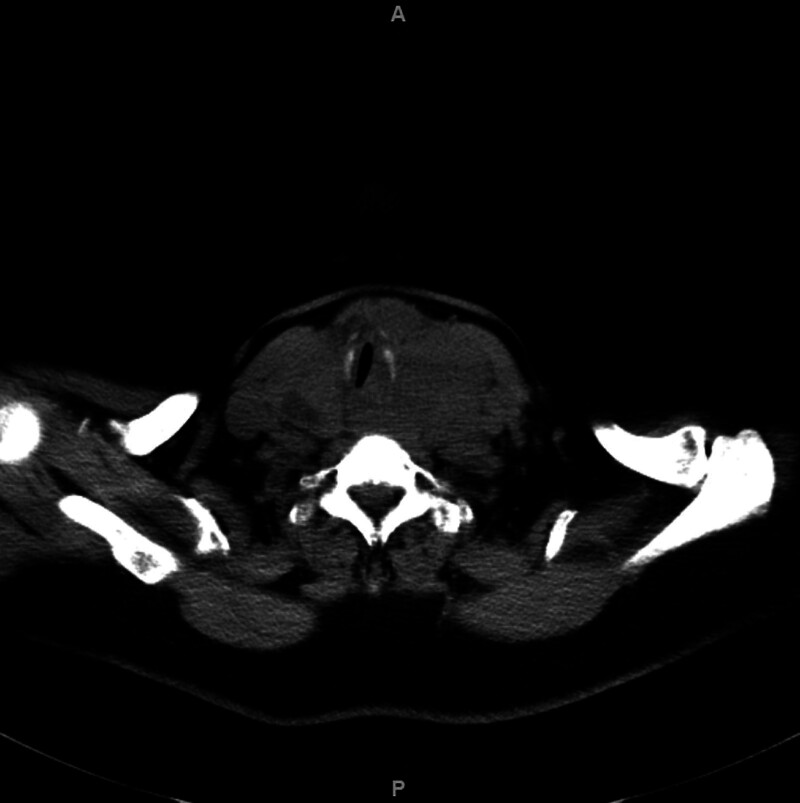
The thyroid gland is significantly enlarged in size, with unevenly reduced density; multiple slightly hypodense nodules are observed; the boundaries are unclear; the trachea is significantly narrowed and pushed to the right side by compression, with the inner diameter of the narrowest part being 0.47 cm.

**Figure 2.**
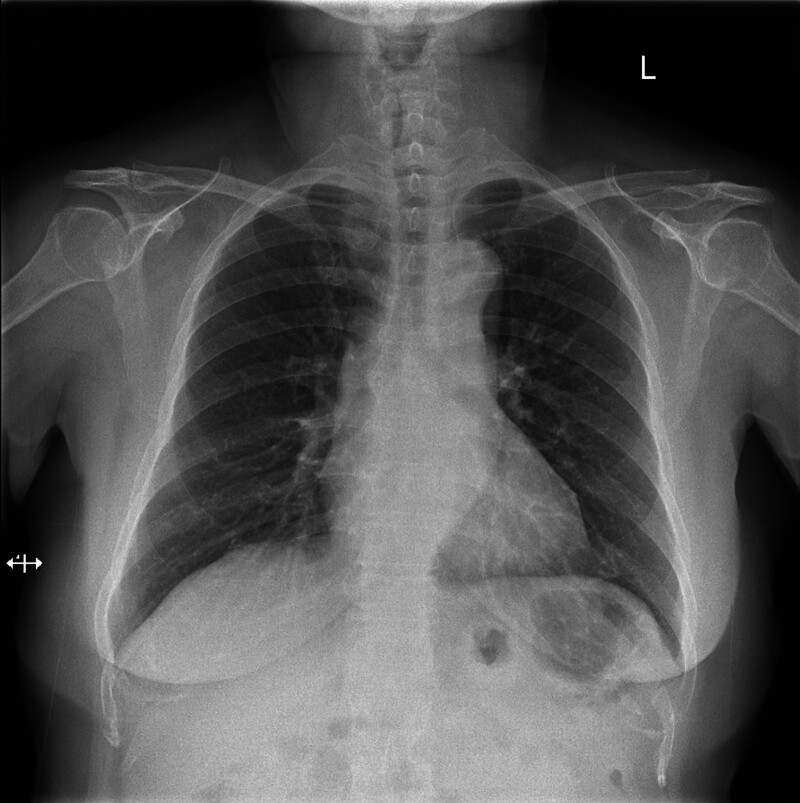
Chest radiograph shows that the enlarged goiter is causing the obvious airway compression and has pushed the airway to the right side.

**Figure 3.**
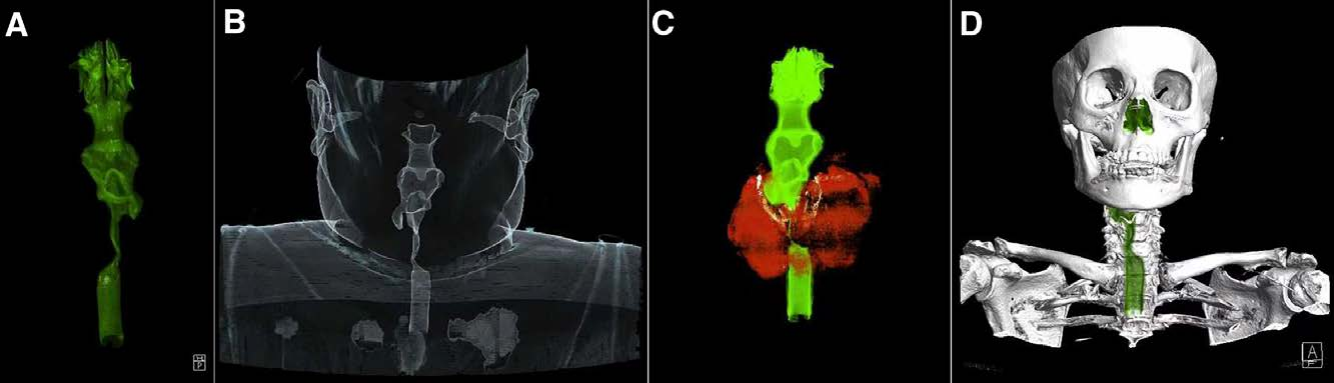
3D reconstruction shows that the goiter has compressed the airway, leading to severe stenosis, and pushed the airway to the right side.

Preoperative neck enhancement CT showed that the thyroid gland was significantly enlarged in size with unevenly reduced density; multiple slightly hypodense nodules were observed with unclear boundaries; and the trachea was significantly narrowed by compression, with the inner diameter of the narrowest part being 0.47 cm (see Figs. 1–3). Multiple, slightly large lymph nodes were observed in the soft tissue spaces of the neck and mediastinum. During preoperative multidisciplinary discussions, it was concluded that the patient’s airway was obviously compressed by the goiter and that routine preoperative orotracheal intubation under guidance is likely to cause difficulty in intubation, airway bleeding, or edema, leading to hypoxia.

After a comprehensive deliberation of options, it was decided to perform VV-ECMO before surgery to assist in ensuring good oxygen supply to the tissues and organs throughout the body, and then perform the goiter resection. After adequate preoperative preparation, the patient was admitted to the operating room on July 29, 2022. Under local anesthesia, the right femoral vein was punctured to place a 21F drainage tube, and the right internal jugular vein was punctured to place a 17F perfusion tube. The patient was heparinized with 5000 U of normal heparin (64 U/kg) and then put on VV-ECMO assistance with a rotational speed of 3500 rpm, a flow rate of 3.7 L/min, and an oxygen concentration of 100%. After ensuring a healthy pulse oximetry and oxygenation index, our repeated attempts at tracheal intubation under visual laryngoscopy failed. We then proceeded to perform a VV-ECMO-assisted total thyroidectomy + tracheal tumor resection + elective cervical lymph node dissection + tracheal reconstruction + tracheal rotational end-to-end anastomosis, which went well. Intraoperative transnasal tracheal intubation was successful, and postoperatively, the patient was weaned off VV-ECMO assistance and transferred to the Department of Critical Care Medicine.

On the 5^th^ postoperative day, the patient developed inspiratory dyspnea after extubation, which was diagnosed as airway stenosis. The patient underwent an emergency tracheotomy, was stabilized postoperatively, transferred back to ENT on the 10^th^ postoperative day, and was discharged from the hospital after 2 weeks of improvement. This patient maintained an oxygen saturation of over 99% during surgery, and all blood gas analysis results were within the ideal range. Detailed intraoperative and postoperative data can be found in Table 1.

**Table 1:** Intraoperative and postoperative parameters in 2 case reports.

Index	Case 1		Case 2	
	Intraoperative ($\bar{X}$)	Postoperative ($\bar{X}$)	Intraoperative ($\bar{X}$)	Postoperative ($\bar{X}$)
PH	7.45	7.38	7.44	7.48
PCO_2_	30.00	34.33	33.27	28
PO_2_	270.23	113.33	297.60	224
SO_2_	99.90	98.30	99.90	98.83
HCT	34.67	32.00	38.67	32.00
HB	106.33	106.67	128.93	107.00
Na^+^	139.67	139.00	139.20	139.00
K^+^	3.79	3.90	3.78	3.63
CI^-^	110.50	111.33	108.03	109.47
Ca^2+^	1.15	1.18	1.26	1.10
Mg^2+^	0.81	0.51	0.71	0.50
Glu	7.05	9.13	7.37	8.00
LAC	1.40	2.47	1.27	1.53
BUN	7.63	7.10	16.33	11.97
BE-ecf	−3.97	−6.07	−2.57	−2.03
BE(B)	−2.33	−3.67	−1.87	−0.83
HCO_3_^-^	20.30	20.20	22.03	20.87
FIO_2_	33.67	40.00	33.93	50.00

### 2.2. Case 2

A 75-year-old woman weighing 70 kg presented to our hospital with chief complaints of undifferentiated thyroid cancer for 4 months and a cough with shortness of breath for 1 month. The patient presented with hemoptysis 4 months ago, and space-occupying lesions were found in the lungs, with a puncture biopsy suggesting a thyroid origin.

A positron emission tomography-computed tomography examination showed that the right lobe of the thyroid gland was enlarged with soft tissue mass formation, accompanied by multiple calcifications and increased glucose metabolism, and was considered to be highly malignant. An ultrasound-guided fine needle aspiration biopsy of solid nodules in the right lobe of the thyroid was performed. Pathology examination results were as follows: needle aspiration + liquid base + wax block of nodule in the lower pole of the right lobe of the thyroid gland: cancer, consistent with thyroid dedifferentiated carcinoma, with a size of 5.1*5*4.9 cm. Admission diagnosis: 1. Thyroid cancer (undifferentiated carcinoma, stage IV); lymph node metastasis (mediastinum, neck); lung metastasis.

**Figure 4.**
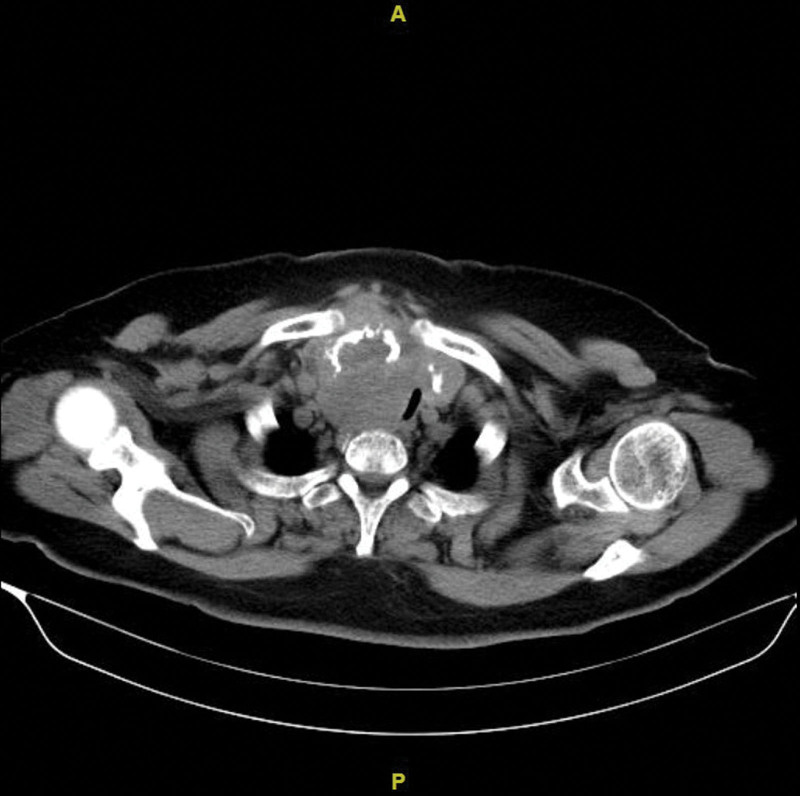
Enlargement of the right lobe of the thyroid gland, with low-density nodular shadows in both lobes, with the right lobe being the larger, approximately 6.8*5.1 cm, accompanied by heterogeneous banded calcifications with unclear boundaries, and the airway is pushed to the left.

**Figure 5.**
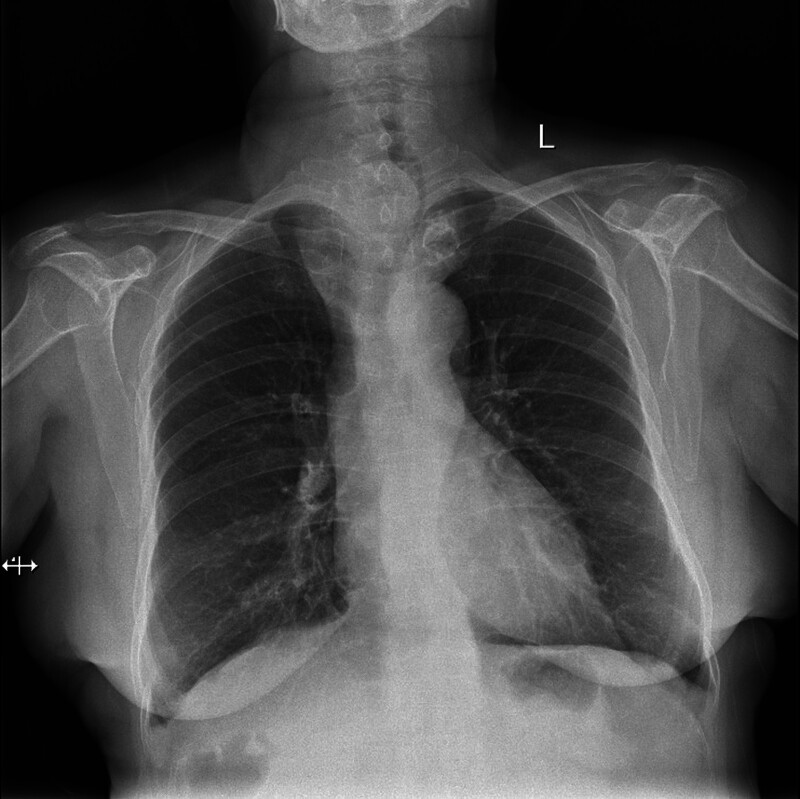
Chest X-ray: quasi-circular iso-hyper-density lesions can be seen on the right side of the upper mediastinum, and the airway is pushed to the left due to airway compression.

**Figure 6.**
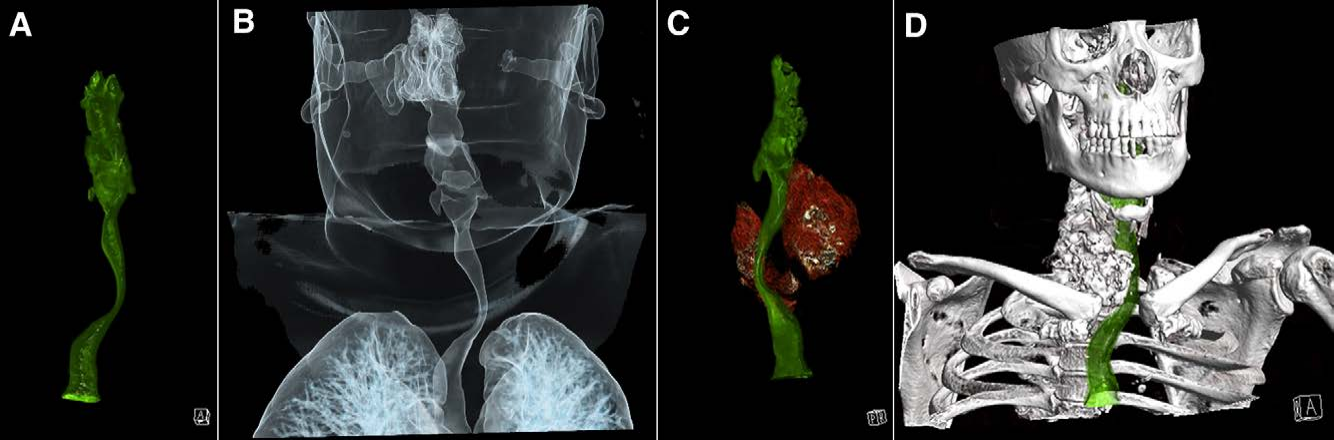
3D reconstruction shows that the right lobe of the thyroid gland is obviously enlarged, and the airway pressure is pushed to the left.

The patient presented with an intermittent cough with progressively aggravated shortness of breath over the past month. The patient was admitted to the general surgery department of the hospital for further treatment. Observations from the preoperative multidisciplinary joint examination were as follows: the patient’s goiter had caused obvious airway stenosis and pushed the airway to the left (Figs. 4–6). Hence, given the difficulty of intubation, it was proposed to make further attempts with a thin tube, and laryngeal edema, intubation failure, and asphyxiation before the operation occurred were not excluded. The patient’s tracheal compression was located at the conventional tracheotomy incision and required full dissection of the tumor to expose the trachea before the incision. As this was difficult to operate on and as there was still a large tumor compressing the trachea in the retrosternal area, the patient was considered for surgical treatment after preoperative VV-ECMO assistance.

After adequate preoperative preparation, the patient was admitted to the operating room. The right femoral vein was punctured under local anesthesia to place a 21F drainage tube for 45 cm. After an ultrasound scan confirmed that the inferior vena cava orifice was well-positioned, the right internal jugular vein was punctured to place a 17F perfusion tube for 15 cm under local anesthesia with ultrasound localization. After administering a loading dose of 4000 U of heparin (57 U/kg), the ECMO line was connected with a blood flow rate of 3 to 5 L/min, a rotation speed of 2000 to 3000 rpm, and an oxygen concentration of 100%. Once the VV-ECMO was functioning well, palliative resection of thyroid cancer (total bilobar thyroidectomy + partial tracheotomy + tracheostomy) was performed under general anesthesia. After tracheotomy, the patient was connected to ventilator-assisted ventilation, and oxygenation was good. The patient was successfully weaned off the ECMO and was transferred to the Department of Intensive Care Medicine for monitoring and further post-operative care.

As per the postoperative pathology investigation, the resection specimen of the bilobar thyroid showed an undifferentiated thyroid carcinoma (right lobe). The patient’s condition was stabilized on the 4th postoperative day and transferred to the general ward. She was discharged on the 10th postoperative day. This patient also maintained an oxygen saturation of over 99% during surgery, and all blood gas analysis results were within the ideal range. Detailed intraoperative and postoperative data can be found in Table 1.

## 3. Discussion

Severe airway stenosis leads to various types of dysfunctional ventilation, such as airway obstruction, massive airway bleeding, and acute tracheal or bronchial lesions, which may lead to severe hypoxia or death. Airway obstruction due to malignancy has been found to be a major cause of severe airway problems.^[3]^ The thyroid gland has a complex anatomical relationship with the pharynx, trachea, and esophagus, and a goiter has a high likelihood of causing airway compression or even invasion of the airway, leading to airway stenosis, progressive dyspnea, hoarseness, dry cough, and hypoxemia, which may lead to respiratory failure or sudden death in severe cases.^[4]^

Surgical resection is the mainstay of treatment, aiming to relieve airway compression and improve the prognosis. In cases of circulatory and respiratory failure due to various causes, extracorporeal membrane oxygenation (ECMO) is used as a bridging technique. This enhances circulation and oxygen supply, which helps further diagnosis and treatment.^[5]^ In both our patients, the goiter enlargement was severe (grade III), compressing the airway, esophagus, carotid artery and vein, and recurrent laryngeal nerve, which could result in respiratory distress and hoarseness.

The extensive stenosis of the airway necessitated this treatment approach since all other options would have inevitably resulted in hypoxic events. Anesthesia works well with the VV-ECMO, and the surgery can progress smoothly without life-threatening situations such as hypoxia. In patients with severe tracheal obstruction due to thyroid or lung tumors, induction of general anesthesia is risky and requires a comprehensive plan. ECMO-assisted anesthesia or surgery is the preferred option to guarantee safe oxygen supply in such patients.^[6–9]^

VV-ECMO is a feasible and safe treatment option for patients with high-risk airway stenosis. In such patients, VV-ECMO should be used aggressively and early during bronchoscopy or intervention.^[10]^ In patients with severe airway stenosis, bronchoscopy-guided examination and treatment may lead to a higher risk of airway edema or bleeding, and VV-ECMO assistance should be considered more aggressively when necessary; however, more research is needed to further substantiate this view.

ECMO therapy is more commonly used in Intensive Care Units, and its use in high-risk surgeries, especially high-risk airway and lung malignancy surgeries, is gaining popularity.^[5,11]^ ECMO assistance can cause associated complications, such as bleeding at the puncture site, infection, and arteriovenous thrombosis, especially when ECMO is performed intraoperatively as anticoagulation is required. Hence, this procedure requires the multidisciplinary collaboration of experienced surgical, anesthesia, and ECMO teams. In this study, neither of the 2 patients exhibited significant abnormal bleeding. The guidelines recommend a heparin sodium dose of 40 to 100 units/kg prior to ECMO initiation. Based on the assessment of bleeding risk, both patients were administered the lowest dose of heparin sodium to mitigate the risk of intraoperative bleeding. Given the heparin coating on the circuit, after the initial heparin sodium loading dose, ECMO typically does not require anticoagulation with heparin sodium for the first 24 hours of operation. If the ECMO is not discontinued after 24 hours, anticoagulation with heparin sodium can be continued based on the levels of APTT (Activated Partial Thromboplastin Time) and ACT (Activated Clotting Time). The important prerequisite for not requiring anticoagulation within the first 24 hours after the initial heparin sodium loading dose is stable flow, as flow is the best anticoagulant. Even though ECMO assistance can lead to complications, it remains an effective and relatively feasible option for treating severe airway stenosis with a high risk of asphyxia.

## 4. Conclusion

Thyroid tumors are common endocrine tumors, with a higher incidence in females than males. Advanced thyroid tumors are very likely to lead to compression or invasion of the trachea. Patients with severe or complete airway stenosis have a high incidence of perioperative hypoxic events and are at great risk during surgery. VV-ECMO provides a safe and convenient mode of oxygen supply for patients with severe airway stenosis caused by thyroid tumors, which reduces the risk during surgery and improves the short- and long-term prognosis.

## Acknowledgments

We would like to acknowledge the hard and dedicated work of all the staff that implemented the intervention and evaluation components of the study.

## Author contributions

**Conceptualization:** Shuang-Long Zhang, Hong-Xun Yuan.

**Data curation:** Shuang-Long Zhang, Wang Zheng, Qi-Feng Zhang, Shi-Lei Zhao, Wei Sun, Li-Na Meng.

**Formal analysis:** Shi-Lei Zhao, Gang Li, Wuyuntana Han.

**Writing – original draft:** Shuang-Long Zhang.

**Writing – review & editing:** Shuang-Long Zhang, Wang Zheng, Qi-Feng Zhang, Gang Li, Wei Sun, Li-Na Meng, Wuyuntana Han, Hong-Xun Yuan.
